# Editorial: Advances in natural macromolecules: chemistry and materials engineering

**DOI:** 10.3389/fchem.2025.1740016

**Published:** 2025-11-21

**Authors:** Qingxin Han, Xiaoyu Guan, Motoki Ueda

**Affiliations:** 1 Institute of Biomass and Function Materials and College of Bioresources Chemistry and Materials Engineering, Shaanxi University of Science and Technology, Xi’an, China; 2 Graduate School of Urban Environmental Sciences, Tokyo Metropolitan University, Tokyo, Japan

**Keywords:** natural macromolecules, chemical design, structural engineering, sustainable materials, low-carbon innovation

Natural macromolecules are at the heart of modern materials innovation. Their structural diversity, biocompatibility, and renewability make them ideal candidates for developing functional materials that align with the goals of green chemistry and sustainable engineering. However, turning these complex biomolecules into reproducible, high-performance materials requires a deep integration of chemistry, structure design, and process control. This Research Topic, *Advances in Natural Macromolecules: Chemistry and Materials Engineering*, highlights how rational chemical design and structural engineering can together generate sustainable, functional, and high-performance materials derived from nature.

A study by Zhang et al. explores protein-based double-network hydrogels designed to reproduce the mechanical and transport behavior of the oral mucosa. By integrating a covalently crosslinked network with a secondary elastin-like polypeptide network, the authors reproduce both the modulus and flexibility of distinct oral regions. The resulting hydrogels reproduced the mechanical properties, hydrophilicity, and permeability of native oral mucosa, providing a realistic platform for studying drug or nanoparticle diffusion across soft tissues. This work demonstrates how controlled network architecture and protein-based design can create reliable biomimetic materials that closely emulate physiological environments.

Extending this understanding to a specific class of structural biomaterials, Guan et al. analyzed how chemical and physical modifications regulate the mechanical behavior of regenerated silk fibroin. By comparing crosslinking, solvent treatment, stretching, and thermal annealing, they clarified how changes in molecular structure and crystallinity control tensile strength and toughness. The study provides clear guidance for tailoring the mechanical performance of silk-based materials through hierarchical structural control.

Hu et al. provided a brief review of naturally derived biomedical polymers such as collagen, gelatin, chitosan, cellulose, alginate, and hyaluronic acid, summarizing recent advances in chemical modification, crosslinking, and composite fabrication. The review highlighted how these strategies improve mechanical strength, biocompatibility, and degradability, and discussed their applications in scaffolds, bioinks, and drug delivery systems. They also emphasized the need for controlled release of bioactive molecules to ensure safe and sustained tissue regeneration. In addition, precise control of scaffold architecture is critical for cell infiltration and nutrient transport, which can be achieved through advanced fabrication methods such as 3D printing and electrospinning.

At a different application Frontier, Alqahtani et al. extended the design philosophy of natural macromolecules to synthetic analogues by fabricating eco-friendly MOF-loaded PVA/PVP nanofibers through electrospinning. The hybrid material shows efficient adsorption of dye pollutants and strong antimicrobial activity, reflecting the same structural and functional principles that guide natural macromolecular systems. It also reflects the versatility of macromolecular platforms for addressing environmental challenges through scalable and low-cost fabrication.

Together, the four contributions in this Research Topic present a coherent progression in the field of natural macromolecule research. They move from fundamental understanding and property optimization to the controlled construction of hierarchical structures and their application in biomedical and environmental contexts. Although the studies address different systems, they share a unified foundation. Molecular design determines function, hierarchical structuring converts molecular interactions into measurable material performance, and engineering translation requires rigorous attention to reproducibility and sustainability.

Chemistry and materials engineering together define the essential framework for understanding and designing biomacromolecules materials. Future progress will rely on establishing predictive relationships between molecular structure and performance, on developing standardized evaluation methods for mechanical and degradation properties, and on integrating life-cycle assessment into material design. The studies in this Research Topic illustrate only a small portion of the advances emerging from the interplay of chemical innovation, structural understanding, and engineering practice, as biomacromolecules materials continue to open new avenues toward a sustainable and low-carbon innovation.

